# TERRA regulates DNA G-quadruplex formation and ATRX recruitment to chromatin

**DOI:** 10.1093/nar/gkac1114

**Published:** 2022-11-28

**Authors:** Ru-Xuan Tsai, Kuo-Chen Fang, Po-Cheng Yang, Yu-Hung Hsieh, I-Tien Chiang, Yunfei Chen, Hun-Goo Lee, Jeannie T Lee, Hsueh-Ping Catherine Chu

**Affiliations:** Institute of Molecular and Cellular Biology, National Taiwan University, No. 1 Sec. 4 Roosevelt Road, Taipei, Taiwan; Institute of Molecular and Cellular Biology, National Taiwan University, No. 1 Sec. 4 Roosevelt Road, Taipei, Taiwan; Institute of Molecular and Cellular Biology, National Taiwan University, No. 1 Sec. 4 Roosevelt Road, Taipei, Taiwan; Institute of Molecular and Cellular Biology, National Taiwan University, No. 1 Sec. 4 Roosevelt Road, Taipei, Taiwan; Institute of Molecular and Cellular Biology, National Taiwan University, No. 1 Sec. 4 Roosevelt Road, Taipei, Taiwan; Institute of Molecular and Cellular Biology, National Taiwan University, No. 1 Sec. 4 Roosevelt Road, Taipei, Taiwan; Department of Molecular Biology, Massachusetts General Hospital, Department of Genetics, Harvard Medical School, Boston, MA 02114, USA; Department of Molecular Biology, Massachusetts General Hospital, Department of Genetics, Harvard Medical School, Boston, MA 02114, USA; Institute of Molecular and Cellular Biology, National Taiwan University, No. 1 Sec. 4 Roosevelt Road, Taipei, Taiwan

## Abstract

The genome consists of non-B-DNA structures such as G-quadruplexes (G4) that are involved in the regulation of genome stability and transcription. Telomeric-repeat containing RNA (TERRA) is capable of folding into G-quadruplex and interacting with chromatin remodeler ATRX. Here we show that TERRA modulates ATRX occupancy on repetitive sequences and over genes, and maintains DNA G-quadruplex structures at TERRA target and non-target sites in mouse embryonic stem cells. TERRA prevents ATRX from binding to subtelomeric regions and represses H3K9me3 formation. G4 ChIP-seq reveals that G4 abundance decreases at accessible chromatin regions, particularly at transcription start sites (TSS) after TERRA depletion; such G4 reduction at TSS is associated with elevated ATRX occupancy and differentially expressed genes. Loss of ATRX alleviates the effect of gene repression caused by TERRA depletion. Immunostaining analyses demonstrate that knockdown of TERRA diminishes DNA G4 signals, whereas silencing ATRX elevates G4 formation. Our results uncover an epigenetic regulation by TERRA that sequesters ATRX and preserves DNA G4 structures.

## INTRODUCTION

DNA forms G-quadruplex (G4) by stacked guanine tetrads held together through Hoogsteen hydrogen bonds (1). G4 DNA is a stable, non-B form DNA structure often carrying a consensus G_≥3_N_1–*x*_G_≥3_N_1–*x*_G_≥3_N_1–*x*_G_≥3_ motif (2,3). By computational analysis, G4 forming sequences have been predicted in genomic regulatory regions and telomeres (4,5). Experimental data show that G4 structures are enriched at active chromatin and nucleosome-depleted regions (6,7), with the most abundant at 1 kb upstream transcription start sites (TSS). Emerging evidence implicates that DNA G4s are associated with highly expressed genes (7,8) and interfering with G4 formation results in dysregulation of transcription (9–14). DNA G4 can be stabilized by several means, including binding to specific transcription factors (15,16) or association with an RNA strand that is forming an R-loop structure (17), which leads to a displaced strand that is rich in guanine sequence for G4 assembly. On the other hand, cellular RNAs contain G4-forming sequences and can fold into RNA G4 (RG4) *in vitro*, but observations of reverse transcription stop profiling combined with dimethyl sulfate treatment show that most endogenous RNAs are globally unfolded in eukaryotic cells (18), indicating that RNA harboring G-rich sequence may be bound by RNA binding proteins and unfolded in most of the cases *in vivo*.

TERRA is a large non-coding RNA that consists of UUAGGG repeats (19) and can fold into G4 structures *in vitro* (20–23) and *in vivo* (24). It is synthesized from subtelomeric regions toward telomeric ends (19,25). Owing to its preferential association with telomeres, the investigation into TERRA function has focused on telomeres (26–30). Through a genomic approach, TERRA is found to bind to chromatin outside of telomeres, and depletion of TERRA by antisense oligos (ASO) alters gene expression in mouse embryonic stem cells (ES) (31). Other studies also reported that introducing a synthetic TERRA RNA oligo (32) or suppressing TERRA transcription by tethering transcription repressors (33) to subtelomeric regions in human cells leads to changes in the transcriptome. These results suggest that TERRA may function as an epigenomic modulator, but the underlying mechanism is poorly understood.

Using iDRiP (identification of direct RNA interacting proteins) proteomic analysis, a large network of TERRA interacting proteins has been identified, including a chromatin remodeler—ATRX (31,34,35). TERRA binds to ATRX and competes with telomeric DNA for ATRX binding *in vitro* (31). TERRA counteracts ATRX for gene expression at several loci: whereas TERRA activates, ATRX represses gene expression (31) in mouse ES cells. Mutations on ATRX gene in humans cause ATRX syndrome (Alpha-thalassemia X-linked intellectual disability), displaying clinical features such as mental retardation, facial, skeletal, and urogenital abnormalities, as well as thalassemia (36). ATRX is able to recognize histone H3 lysine9 trimethylation (H3K9me3) (37,38) and maintains heterochromatin silencing by facilitating histone H3.3 recruitment and H3K9me3 formation (39). Biochemical analyses indicate that ATRX contains G4 DNA binding ability *in vitro* (40) and enables the unwinding of Hoogsteen hydrogen bonds within DNA triplex structures (34,41). Furthermore, loss of ATRX in mouse and human cells leads to accumulation of G4 structures *in vivo* (42,43), denoting a link between ATRX and G4 maintenance.

Here we investigate the function of TERRA RNA in modulating chromatin status on a genome-wide scale. Interestingly, we find that TERRA promotes G4 formation at transcription start sites (TSS), while it prevents ATRX from loading onto TSS and subtelomeric regions. Our data uncover the epigenetic regulation by TERRA, which works beyond telomere biology and regulates DNA G4 structures across the genome.

## MATERIALS AND METHODS

### LNA transfection

Mouse 16.7 embryonic stem cells (ESCs) were grown until 70% confluency and trypsinized. After removing feeder cells, ES cells were maintained on gelatinized dishes with feeder-conditioned medium. Followed by one passage after feeder removal, LNA transfection was performed using a mouse ES cell nucleofector kit (Lonza, Cat#VPH-1001). Cells were fed with fresh medium 3 h before transfection. A total of 3 × 10^6^ mESCs were transfected with LNA gapmer oligos at a concentration of 4 μM in 100 μl nucleofector solution using A-23 program. Cells were plated onto gelatinized 6-well plates with 2 ml of half feeder-conditioned medium (medium from feeders grown in mES medium for 12 h) and half fresh mESCs medium. Anti-sense TERRA (5′-TAACCCTAACCCTAAC-3′) and control (scramble) LNA (5′-CACGTCTATACACCAC-3′) gapmers were purchased from Qiagen with modified LNA bases and phosphothiolated backbone modification.

### Chromatin immunoprecipitation sequencing (ChIP-seq)

6.5 × 10^6^ cells were fixed in ES medium containing 1% (v/v) formaldehyde for 5 min at room temperature (RT). The reaction was quenched by adding 1/10 volume of 1.25 M glycine and incubated at RT for 5 min. Cells were washed with ice-cold PBS and lysed in 0.5 ml Buffer 1 (50 mM HEPES, 150 mM NaCl, 1 mM EDTA (pH 8.0), 0.5% NP-40, 0.25% Triton X-100) supplemented with 2× complete EDTA-free Protease Inhibitor Cocktails (PIC, Roche, Cat#04693132001), and incubated for 10 min with rotation at 4°C. Cells were pelleted by centrifugation at 21,100 *g* for 10 min at 4°C, resuspended in Buffer 2 (200 mM NaCl, 5 mM EDTA, 2.5 mM EGTA, 10 mM Tris pH 8.0) supplemented with 2× PIC, and rotated at 4°C for 10 min. After centrifugation, cells were resuspended in Buffer 3 (5 mM EDTA, 2.5 mM EGTA, 10 mM Tris pH 8.0, and freshly added 2× PIC), mixing on a rotator for 10 min at 4°C. 30 μl of 10% *N*-lauroylsarcosine was added to lysates and incubated on a rotator for 10 min at 4°C. For sonication, 130 μl of samples were loaded into Covaris microtube (6 × 16 mm), using the program with 12-height-level for water-bath, at 4–7°C, 5% duty cycle, intensity 4 and 200 bursts per cycle for 10 min. After sonication, the insoluble material was removed by centrifugation at 4°C. The fragmented DNA size is around 200–500 bp for ATRX and H3K9me3 ChIP-seq. For immunoprecipitation, an equal volume of 2× IP buffer (2% Triton X-100, 300 mM NaCl, 30 mM Tris–HCl pH 6.8, 1× PIC) was added into chromatin lysates containing 5 μg chromatin, and incubated with 2 μg of antibodies at 4°C overnight on a rotator. 20 μl of Protein G beads (washed with Buffer 1 twice and resuspended in Buffer 1) were added into lysates and then rotated at 4°C for 2 h. Beads were washed with RIPA-I buffer (50 mM HEPES, 10 mM EDTA (pH 8.0), 0.5% Sodium Deoxycholate, 0.5% NP40 and 500 mM NaCl) 3 times and RIPA-II (50 mM HEPES, 10 mM EDTA, 0.5% sodium deoxycholate, 0.5% NP-40 and 50 mM NaCl) 3 times, 5 min each at 4°C with rotation. Beads were resuspended in 100 μl TES buffer (10 mM EDTA, 1% SDS, and 50 mM Tris pH 8.0) at 65°C for 15 min for elution. 1 μl of 20 mg/ml RNase A was added to each sample and incubated at 37°C for 20 min. Samples were further incubated with 5 μl of 20 mg/ml Proteinase K at 55°C overnight. After reverse crosslinking, DNA was purified using a Plus DNA Clean / Extraction Kit. Antibodies against IgG (Cell Signaling, Cat#2729S), ATRX (Santa Cruz, Cat#sc-15408), and H3K9me3 (Abcam, Cat#8898) were used. For G4 ChIP, most steps were the same as the ChIP protocol described above. Few steps were modified: Cells were resuspended in Buffer 1 for 30 min at 4°C. The sonication time was extended to 20 min to reach the fragmentation size below 250 bp. RIPA-I buffer(50 mM HEPES, 10 mM EDTA (pH 8.0), 0.5% Sodium Deoxycholate, 0.5% NP40 and 500 mM LiCl). RIPA-II (50 mM HEPES, 10 mM EDTA, 0.5% Sodium Deoxycholate, 0.5% NP-40 and 50 mM LiCl). BG4 (Absolute antibody, #Ab00174-1.1) antibody was used. Eluted DNA was subjected to library construction using NEBNext Ultra II DNA Library Prep kit (NEB, Cat#E7645S) for Illumina. Paired-end 150bp reads were obtained by Illumina HiSeq 4000 or NovaSeq systems.

### Data analysis

ChIP-seq and ATAC-seq raw FASTQ reads were trimmed by Trim-galore (v0.5.0) (https://github.com/FelixKrueger/TrimGalore) with parameters: –illumina –fastq -q 30. Reads containing telomeric repeats (Supplementary Figure S1C) were counted by BBDuk (v38.23) (https://jgi.doe.gov/data-and-tools/bbtools/bb-tools-user-guide/) with parameters: K = 24, literal=TTAGGGTTAGGGTTAGGGTTAGGG. Trimmed reads were aligned to GRCm38/mm10 reference genome using bowtie2 aligner (v2.3.4.1) (44) with the default setting. Repeat Enrichment Estimator (45) was utilized to analyze the enrichment of repetitive elements in ATRX and H3K9me3 ChIP-seq data. BAM files were sorted and deduplicated with SAMTools (v1.9) (46). Enriched peaks were determined using MACS1.4 or MACS2 (v2.1.2) (47) (https://github.com/macs3-project/MACS) with cutoff values: –pvalue 0.01. ATAC-seq peaks were called using MACS2 with -*P-*value < 0.01 cutoff, and without input treatment normalizing (exclude parameter -t option). Peak bed files and wig files generated from MACS1.4 were used for CEAS (Cis-regulatory Element Annotation System, v1.0.2) (48) analysis to assess ChIP enrichment on genomic features such as specific chromosome, promoter, gene body, exon, intron, and intergenic regions. Bedtools intersect (v2.3.0) (49) was used to determine overlapping regions of two interested data. ATRX binding sites within subtelomeric genes were generated from the overlapping regions of ATRX peaks called by MACS1.4 in TERRA KD mESCs and the bed file of subtelomeric transcripts. Active transcription starting sites were obtained from H3K4me3 peaks called by MACS2. ATRX peaks on active TSS regions in TERRA KD mESCs (Figure 6D) were defined by the common regions of ATRX peaks and H3K4me3-enriched TSS. G4 peaks on open-chromatin regions used in Supplementary Figure S4B were generated using common peaks of scramble KD and TERRA KD ATAC-seq called by MACS2 and scramble KD G4 peaks called by MACS1.4. Bedtools fastaFromBed (v2.3.0) (49) converted a region to fasta information for motif sequence analysis, which was conducted using MEME Suite (v5.3.2) (50). Bigwig files were generated using DeepTools (v3.1.1) (51) bamCoverage. Normalized bigwig files and bedgraph files were normalized to inputs by DeepTools (v3.1.1) (51) bamCompare with parameters: –operation subtract, –centerReads, –normalizeUsing CPM, –scaleFactorsMethod None. ATAC-seq coverage was generated by DeepTools (v3.1.1) (51) bamCoverage. Coverages were visualized in Integrative Genomics Viewer (v2.10.3) (52). For all genomic views in the figures, the coverages of ATRX, H3K9me3, and G4 ChIP-seq data were normalized with input files and total mapped reads, with the exception in Figure 3E, in which G4 ChIP and input files were only normalized with total mapped reads. Normalized bedgraph files were used to plot metagene profiles by HomerTools (v4.1) (53), and few outliers with extremely high density were removed in the analysis of G4 coverage at TERRA binding sites. HomerTools annotatePeaks.pl was used to identify associated gene ID, gene name, and feature of ChIP-enriched peaks.

Gene ontology analysis of ATRX peaks on promoter regions in TERRA KD or scramble KD was performed using DAVID Bioinformatics Resources (54,55). Bigwig files normalized to input files using log2 ratio were applied to heatmap plots, which were generated by DeepTools (v3.1.1) (51) computeMatrix and plotHeatmap.

Heatmap clusters were grouped by *k*-means algorithm. Significant correlation between two heatmap clusters was calculated based on the cumulative distribution function (CDF) of the hypergeometric distribution (https://systems.crump.ucla.edu/hypergeometric/); Analyses of over or under enrichment by hypergeometric distribution for ATRX clusters and G4 clusters with parameters: population size (*N*): total number of genes in all clusters, number of successes in the population (*M*): number of genes in a G4 cluster, sample size (s): number of genes in an ATRX cluster, number of successes (*k*): number of genes presented in both G4 cluster and ATRX cluster. Analyses of ATRX or G4 clusters compared to TERRA-regulated genes (DEGs) were performed with parameters: population size (N): total number of genes in all ATRX or G4 clusters, number of successes in the population (*M*): number of TERRA KD upregulated or downregulated genes, sample size (*s*): number of genes in an ATRX or G4 cluster, number of successes (*k*): number of TERRA KD upregulated or downregulated genes in an ATRX or G4 cluster.

TERRA KD RNA-seq analysis was performed as described previously (31). Differentially expressed genes were identified by *P* values <0.05 using DESeq2 (56).

### ATAC-seq

ATAC-seq was performed using the ATAC-seq kit (Active Motif, Cat#53150) according to the manufacturer's instructions. For each ATAC-seq reaction, 5 $ \times$ 10^4^ to 1 $ \times$ 10^5^ cells were used in the following procedures. Cells were trypsinized, harvested, and washed with 100 μl of ice-cold PBS, and centrifuged at 500 *g* for 5 min at 4°C. The supernatant was removed and cell pellets were resuspended in 100 μl of ice-cold ATAC lysis Buffer. Cell pellets were fully resuspended by pipetting, and samples were spun at 500 *g* for 10 min at 4°C. 50 μl of Tagmentation Master Mix was added into the cell lysates (25 μl of 2× tagmentation buffer, 2 μl of 10× PBS, 0.5 μl of 0.5% digitonin, 0.5 μl of 10% Tween 20, 12 μl of high purity water and 10 μl of assemble transposons). Tagmentation reaction was carried out at 37°C for 30 min. 250 μl of DNA purification binding buffer and 5 μl of 3 M sodium acetate were added to each sample. DNA was purified using DNA purification columns. For the ATAC-seq library construction, dual indexes were used for the amplification step. Tagmented DNA was mixed with PCR mixture (33.5 μl of Tagmented DNA, 2.5 μl of i7 Indexed Primer (25 μM), 2.5 μl of i5 Indexed Primer (25 μM), 1 μl of dNTPs (10 mM), 10 μl of 5× Q5 Reaction Buffer, 0.5 μl of Q5 Polymerase (2 U/μl)), and PCR reaction was carried out in thermocycler with the following program: 72°C for 5 min, 98°C for 30 min; 10 cycles of 98°C for 10 s, 63°C for 30 s and 72°C for 1 min; finally, hold at 10°C. After amplification, DNA was size-selected by SPRI beads.

### ATRX knockdown

Mouse ES cells were transfected with siRNA using Lipofectamine RNAiMax. 5 × 10^4^ ES cells were transfected with 20 nM of siRNA for 48 h in feeder-cultured conditions, and the siRNA-contained medium was renewed after 24 h of transfection. The transfected ES cells were collected after removing MEF feeders. Two siRNAs (Thermo Fisher Scientific, Silencer^®^ Select, Cat#s76136, and Cat#s76137) against ATRX and one control siRNA (Thermo Fisher Scientific, Silencer^®^ Select, Cat#4390843) were used. For double knockdown of ATRX and TERRA, mouse ES cells were transfected with siATRX or control siRNA using Lipofectamine RNAiMax in the feeder-cultured condition for 36 h, and then feeders were removed. After culturing in the feeder-free condition (supplied with feeder-conditioned medium) for 12 h, cells were transfected with antisense TERRA LNA oligos or control LNA oligos in combination of siRNAs using nucleofection. Cells were collected after 12 h for RNA extraction.

### G4 ligand treatment

Mouse ES cells were grown until 70% confluency with MEF feeder cells, trypsinized, and then MEFs were removed. ES cells were cultured in the feeder-conditioned medium and plated onto gelatinized plates for 12 h. To induce G-quadruplex formation, cells were washed with DPBS (Gibco, Cat#14190-136) and treated with 0.4 μM of CX-5461 (AbMole, Cat#M2293) in the feeder-conditioned medium for 4 h.

### ChIP quantitative real-time PCR

Quantitative PCR was performed using iQ SYBR Green Supermix (Bio-Rad, Cat#1708882). ChIP-qPCR condition for telomeric repeat DNA: a cycle of 95°C for 3 min, followed by 40 cycles of 95°C for 15 s and 55°C for 2 min. ChIP-qPCR condition for rDNA: a cycle of 95°C for 3 min, followed by 40 cycles of 95°C for 10 s and 58°C for 1 min. (Telomeric repeat Forward 5′-GGTTTTTGAGGGTGAGGGTGAGGGTGAGGGTGAGGGT-3′, Reverse 5′-TCCCGACTATCCCTATCCCTATCCCTATCCCTATCCCTA-3′), (18S Forward 5′-TAGAGGGACAAGTGGCGTTCAG-3′, Reverse-5′-ATCCAATCGGTAGTAGCGACGG-3′), (Core Forward 5′-AGTTGTTCCTTTGAGGTCCGGT-3′, Reverse-5′-CAGCCTTAAATCGAAAGGGTCT-3′), (Pro Forward 5′-AGATAGGTACTGACACGCTGTCC-3′, Reverse-5′-ACAGCTTCAGGCACCGCGAC-3′), (ETS Forward 5′-CCAAGTGTTCATGCCACGTG-3′, Reverse-5′-CGAGCGACTGCCACAAAAA-3′), (ITS Forward 5′-CCGGCTTGCCCGATTT-3′, Reverse-5′-GCCAGCAGGAACGAAACG-3′), (28S Forward 5′-CAGGAGGTGTCAGAAAAGTTACCAC-3′, Reverse-5′-CGTTCAGTCATAATCCCACAGATGG-3′), (T6-T8 Forward 5′-CCTTTACTCTTCCCCACAGCGATTC-3′, Reverse-5′-CGGGACACTTTCGGACATCTGG-3′).

### Northern blot

Oligo probes were end-labeled using T4 polynucleotide kinase. RNA was extracted using TRIzol followed by acid phenol extraction. Total RNA (5 μg) was loaded in each lane. Hybridization was carried out at 42°C overnight using Church buffer and washed at 42°C three times with 2× SSC buffer (2× SSC, 0.5% SDS). GAPDH probe: 5′-GTAGACCCACGACATACTCAGCACCGGCCTCACCCCATT-3′. TERRA probe: 5′-(TAACCC)_5_–3′.

### Statistical analysis

Statistical parameters including the exact value of n, median (m), standard deviation (SD), standard errors (S.E.), Pearson's *R* correlation test, Student's *t* test, Mann–Whitney *U* test, Wilcoxon paired signed-rank test, Kolmogorov–Smirnov test, hypergeometric distribution test, and statistical significance were reported in the Figures and the Figure legends.

### ES cell culture

Mouse 16.7 (cas/mus hybrid, 40, XX, RRID:CVCL_A6UI) (57) embryonic stem cells (ESCs) were all tested and confirmed that they were free from mycoplasma contamination. Mouse ESCs were cultured in ES medium (500 ml DMEM with the addition of 1 ml of β-mercaptoethanol, 6 ml of MEM NEAA, 90 ml of FBS, and 500 μl of LIF) on feeders. Feeders were prepared from primary mouse embryonic fibroblasts treated with 5 μg/ml mitomycin C for 2 h to arrest the cell cycle.

## TERRA RNA-FISH

Cells were cytospun and air dried. Cells were washed with ice-cold PBS and treated with CSKT buffer (10 mM PIPES, pH6.8, 100 mM NaCl, 3 mM MgCl_2_, 0.3 M Sucrose, 0.5% Triton X-100, adjust pH to 6.8) for 2 min at 4°C, fixed in 4% paraformaldehyde for 10 min at RT, stored in 70% ethanol at -20°C. Before hybridization, cells were washed twice with 1× PBS for 5 min at 4°C, subsequently dehydrated in 70%, 80%, 90% and 100% of ethanol for 2 min at room temperature, and then air-dried. The probes (TERRA, (TAACCC)7-Alexa488-3′) were diluted in hybridization buffer (2× SSC, 2 mg/ml BSA, 10% dextran sulfate, and 50% formamide) at the final concentration of 0.5 pmol/ml and denatured at 80°C for 5 min and 37°C for 10 min. Cells were incubated with probes at 42°C overnight. After incubation, slides were washed with 2× SSC / 50% formamide twice and 2× SSC three times, 5 min each at 44°C. Cells were mounted with ProLong Gold Antifade solution with DAPI and stored at 4°C, protected from light. All buffers were prepared using DEPC-treated water.

### DNA G4 immunostaining and quantification

ES cells were cytospun onto slides, CSKT treated, fixed in 4% paraformaldehyde, and stored in 70% EtOH at –20°C as described above. Cells were dehydrated in 70%, 80%, 90% and 100% of ethanol for 2 min at room temperature, and then air-dried. Cells were incubated with 400 μg/ml of RNase A at 37°C for 20 min, and washed twice with 1× PBS for 5 min at room temperature. Cells were incubated with blocking solution (1% BSA/PBS with 1 mM EDTA, pH 8.0) at RT for 30 min. Following blocking, cells were incubated with primary antibodies (BG4, Absolute antibody, Cat#Ab00174-1.1, 1: 200 dilution) in blocking solution at RT for 2 h. After incubation, slides were washed with 0.2% PBST (0.2% Tween-20 in 1× PBS) three times, 5 min each. Cells were incubated with secondary antibodies in blocking solution at RT for 1 h and then washed by 0.2% PBST three times, 5 min each. Cells were mounted with ProLong Gold Antifade solution with DAPI and stored at 4°C, protected from light. For BG4-Flag immunostaining, the steps were the same described above except for the antibody incubation. Slides were incubated with BG4-Flag antibody (Merck Millipore, Cat#MABE917, 1:200 dilution) for 2 h at room temperature and washed three times with 0.2% PBST at room temperature. Slides were incubated with rabbit anti-Flag antibody (Merck Millipore, Cat#F7425, 1:100 dilution) incubation for 2 h at room temperature. G4 staining for TERRA KD and CX-5461 experiments used BG4 antibodies. G4 staining for ATRX KD experiments used either BG4-Flag or BG4 antibodies. Similar results were obtained using BG4-Flag or BG4 antibodies. For quantification of fluorescent intensity, 3D images were taken using an Olympus IX83 microscope and Hamamatsu C13440 digital camera. All images in the same experiments were captured with the same exposure time. 3D images were projected to 2D using the maxima intensity projection function, and G4 intensity within nuclei selected by DAPI staining was calculated using CellSens (Olympus) software. Statistical significance determined by the Mann–Whitney test was analyzed by GraphPad Prism.

### RT-qPCR

Total RNA from knockdown samples was extracted using TRIzol^®^ Reagent according to the manufacturer's instruction. cDNA was synthesized by SuperScript IV reverse transcriptase (Thermo Fisher) using 1 μg of RNA. Primers for quantitative PCR: ATRX Forward 5′-TTTCCTGTCTCCTGGCACTA-3′, Reverse 5′-GGGTGTGAGCCTAAAAACCA; Nfe2l2 Forward 5′-ATGGACTTGGAGTTGCCACC-3′, Reverse 5′-CCTGTTCCTTCTGGAGTTGCT; Shroom4 Forward 5′-GTCCCCTTTGCACAACAACC, Reverse 5′-CTCGGTTTGGGTGTCCATCA; Gm32856 Forward 5′-GGATTCACGTCAGCACTCACC-3′, Reverse 5′-ATCAGAAGCAGCCAGGGAACT; Erdr1 Forward 5′-CACAGTGATGTCACCCACGA-3′, Reverse 5′-GTGAGAATCGCTCCGTCCTG-3′; Smg7 Forward 5′-GTCCCAGATGAGGACCGAAA-3′, Reverse 5′-CGATTCTTGGCTTGGCCTTG-3′; Itpr3 Forward 5′-AGCCAAGCAGACTAAACAGGAC, Reverse 5′-GCCGCTTGTTCACAGTTAAGTA-3′.

### Nuclear fractionation

The pellet containing 2 × 10^6^ mES cells was resuspended in 1 ml Buffer A (10 mM HEPES, 5 mM MgCl_2_, and 0.25 M sucrose with freshly added 0.5 mM DTT, 0.2 mM PMSF and 2× protease inhibitors). The cells were centrifuged at 2500 *g* at 4°C for 5 min and the supernatant was removed. The cell pellet was resuspended in 250 μl Buffer A containing 0.2% NP40 and lysed on ice for 5 min, and centrifuged at 2500 *g* at 4°C for 5 min. The supernatant (cytosolic fraction) was collected and the pellet was washed with 400 μl Buffer A without detergent. The cells were centrifuged at 2500 x g at 4°C for 5 min and the supernatant was discarded. The nuclear pellet was resuspended in 160 μl Buffer B (10 mM HEPES, 0.1 mM EDTA, 1.5 mM MgCl_2_, 300 mM NaCl and 25% glycerol) and incubated on ice for 30 min, and then centrifuged at 18 000 *g* at 4°C for 20 min. The supernatant was collected (nuclear soluble). The pellet was resuspended in 50 μl Benzonase Buffer (25 mM Tris–HCl pH 7.5, 20 mM NaCl, 10% glycerol and 0.4 μl Benzonase), incubated on ice for 60 min, and 50 μl of 2 M KCl with 0.4 mM EDTA was added (final concentration reached to 1 M KCl) and incubated on ice for 10 min (chromatin bound). To reduce the salt concentration, 400 μl of 2× sample buffer was added to chromatin bound samples. 5% of each fraction was loaded for western blotting analysis.

### Western blotting analysis

ES cells were lysed in 2× sample buffer (0.1 M Tris pH 6.8, 2% SDS, 20% glycerol, 0.2 M DTT and 0.017% bromophenol Blue) supplemented with 2× protease inhibitor cocktails for 10 min on ice and heated at 95°C for 5 min. Samples were stored at -30°C or used directly. Antibodies: ATRX (mouse monoclonal), Santa Cruz, Cat#sc-55584, 1:2000 dilution. α1c Tubulin (mouse monoclonal), Santa Cruz, Cat#sc-134239, 1: 2000 dilution. ATRX (polyclonal), MyBioSource MBS7045314, 1:2000 dilution. H3 (rabbit monoclonal), abcam, Cat#ab-1791, 1:8000 dilution. GAPDH (mouse monoclonal), abcam, Cat#ab-8245, 1:5000 dilution.

## RESULTS

### TERRA regulates ATRX binding on chromatin

**Figure 1.**
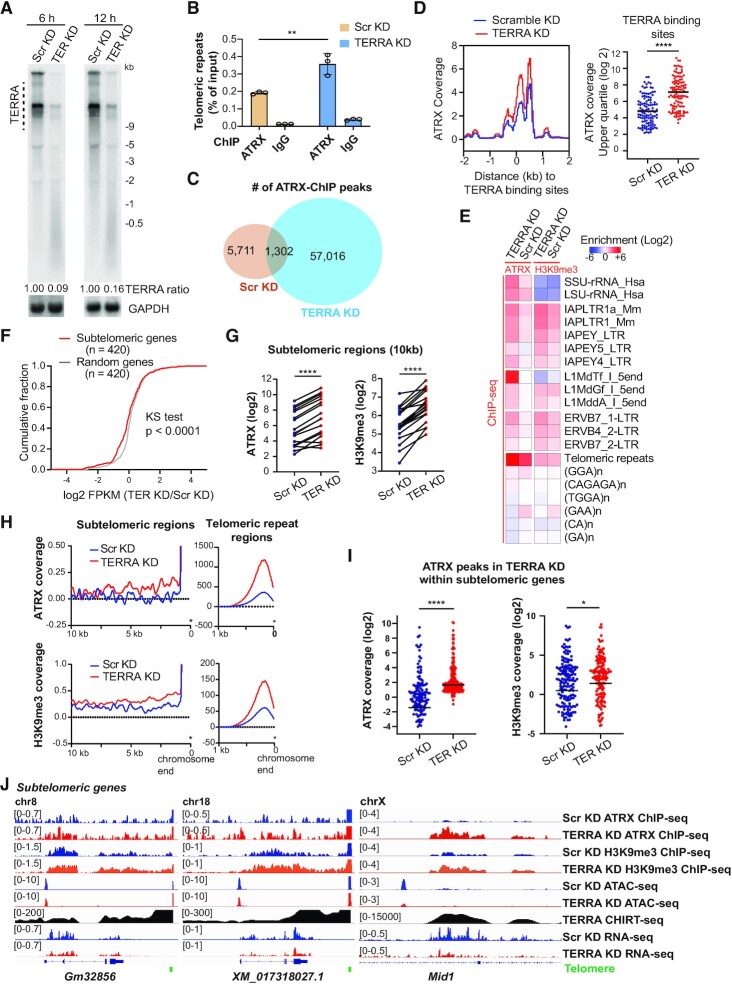
Loss of TERRA promotes ATRX binding to subtelomeric regions, repetitive sequences, and TERRA target sites in mES cells. (**A**) Northern blot analysis for TERRA in mouse ES cells after scramble knockdown (Scr KD) or TERRA KD by ASOs transfection for 6 hrs or 12 hrs. GAPDH, loading control. (**B**) ChIP-qPCR analysis showing the occupancies of ATRX on telomeric repeats after TERRA depletion for 12 hrs in mouse ES cells. Bars, mean ± SD. ** *P* < 0.01, Student's t-test. (**C**) Venn diagram showing the number of intersected peaks for ATRX ChIP-seq between Scr KD and TERRA KD cells. (**D**) TERRA depletion increases ATRX occupancy at TERRA binding sites. Metagene profile (left) of ATRX coverage at TERRA binding sites upon TERRA depletion. Dot plot (right) of ATRX coverages (upper quartile) at TERRA binding sites obtained from TERRA CHIRT-seq. Bars, mean. **** *P* < 0.0001, by Wilcoxon paired signed-rank test. (**E**) Log_2_ fold change (normalized to input) represents the enrichment of ATRX and H3K9me3 on repetitive sequences. (**F**) Cumulative fraction plots for subtelomeric genes (n = 420) and random genes (n = 420) showing log2 fold changes of FPKM (TERRA KD/Scr KD) from RNA-seq data after TERRA KD for 12 h. *P* value, Kolmogorov–Smirnov (KS) test. (**G**) Dot plots showing ATRX and H3K9me3 coverages in subtelomeric regions (10 kb from the pure telomeric sequences at the end of each chromosome). Lines connect the same chromosome ends in Scr and TERRA KD. **** *P* < 0.0001, by Wilcoxon paired signed-rank test. (**H**) Metagene analyses of ATRX and H3K9me3 in subtelomeric regions that are 10 kb away from the end of chromosomes (left). The signals from the pure telomeric tracks at the end of chromosomes (right). (**I**) Dot plots showing ATRX and H3K9me3 coverages on ATRX binding sites within subtelomeric genes. ATRX binding sites were selected from TERRA knockdown cells. Bars, mean. **** *P* < 0.0001, * *P* < 0.05 by Wilcoxon paired signed-rank test. (**J**) Genomic tracks showing increased ATRX and H3K9me3 occupancies on subtelomeric genes after TERRA depletion. ATRX and H3K9me3 coverage files were normalized with the input and total mapped reads. ATAC-seq and RNA-seq displaying chromatin accessibility and gene expression respectively. TERRA CHIRT-seq representing the TERRA binding sites.

TERRA depletion leads to dysregulation of ATRX localization in mouse ES cells shown in the immunostaining study (31). To elucidate how TERRA modulates ATRX distribution on a genome-wide scale, we performed ATRX ChIP-seq (chromatin immunoprecipitation sequencing) after TERRA depletion for 12 h in mouse ES cells. TERRA was depleted using antisense oligonucleotides (ASO) with locked nucleic acids (LNA) chemistry and a gapmer design to enable RNase-H mediated degradation of TERRA RNA *in vivo*. ASO transfection resulted in the downregulation of TERRA transcripts determined by Northern blotting and RNA FISH (Figure 1A, Supplementary Figure S1A). The amount of total ATRX protein was not altered after TERRA depletion, but ATRX in the chromatin bound fraction was increased (Supplementary Figure S1B). ChIP-qPCR for ATRX abundance on telomeric repeats was significantly increased (Figure 1B) after TERRA knockdown. Consistent with the qPCR result, ATRX ChIP-seq data reported that the number of the read counts containing telomeric repeats was higher in TERRA knockdown (KD) compared to the control (scramble KD) (Supplementary Figure S1C). ChIP-seq data were reproduced with a high correlation between two biological replicates (Supplementary Figure S1D). Remarkably, the total number of ATRX peaks was higher in TERRA knockdown cells in comparison to the control (Figure 1C), indicating that TERRA impacts ATRX recruitment on chromatin. Moreover, metagene profiles demonstrated that ATRX occupancy was elevated at TERRA binding sites (31) (Figure 1D) and the telomeric repeat motif upon TERRA depletion (Supplementary Figure S1E), supporting the idea that TERRA prevents ATRX from binding on telomeric repeat sequences and TERRA target sites.

### TERRA depletion results in dysregulation of ATRX occupancy on repetitive sequences

Owing to ATRX preferentially binding on heterochromatin and repetitive sequences (40), we assessed repeat-containing reads of ATRX ChIP-seq data using Repeat Enrichment Estimator (45). The result revealed that TERRA depletion exhibited an increase in ATRX occupancy on rDNA repeats (SSU-rRNA and LSU-rRNA), intracisternal A particle (IAP) retrotransposons, long interspersed nuclear element-1 (LINE-1 transposable elements/L1), and betaretroviral ERV elements (ERVB) (Figure 1E). In agreement with ATRX ChIP-seq, qPCR data showed an elevated enrichment of ATRX at rDNA loci including the regions of the core and 18S in TERRA KD cells compared to scramble KD cells (Supplementary Figure S1F). We also conducted H3K9me3 ChIP-seq upon TERRA depletion. Correspondingly, the abundance of H3K9me3 on IAP and ERVB was also increased in TERRA KD cells (Figure 1E). The data indicate that TERRA regulates ATRX loading and H3K9me3 formation on a subset of repetitive sequences.

### TERRA prevents ATRX recruitment and trimethylation of histone H3K9 in subtelomeric regions

Analyses of RNA-seq data demonstrated that TERRA depletion altered gene expression in mouse ES cells (31), particularly in genes with TERRA target sites and near the end of chromosomes. The cumulative distribution plot showed a subtle left shift of the curve for subtelomeric genes to the downregulation side after TERRA depletion, in comparison to the same number of random genes selected from mouse ES cells (Figure 1F), indicating that subtelomeric genes tend to be suppressed after TERRA depletion. Analyses of the subtelomeric regions, which locate within 10 kb from the end of each chromosome, revealed that TERRA depletion increased the ATRX and H3K9me3 occupancy at nearly all chromosome ends (Figure 1G and H, Supplementary Figure S1G). We further assessed ATRX and H3K9me3 enrichments on 20 subtelomeric transcripts that are transcribed from the end of each chromosome. Dot plots and metagene profiles displayed that the increased abundance of ATRX was accompanied with elevated H3K9 trimethylation in the subtelomeric genes in TERRA KD cells (Figure 1I, Supplementary Figure S1H). The heatmap of the subtelomeric regions that are 100 kb away from telomeres also showed increased ATRX and H3K9me3 occupancies after TERRA depletion (Supplementary Figure S1G). In agreement with this result, the genome browser views presented that ATRX and H3K9me3 occupancies were increased in subtelomeric genes including *Mid1* on sex chromosomes, *XM_017318027.1* on chromosome 18, and *Gm32856* on chromosome 8 after TERRA depletion (Figure 1J). Notably, the chromatin accessibility determined by ATAC-seq (Assay for Transposase-Accessible Chromatin using sequencing) on these genes was reduced in TERRA KD cells (Figure 1J), indicating that TERRA ablation leads to chromatin compaction in the subtelomeric regions. Collectively, these results imply that TERRA prevents ATRX from binding to subtelomeric regions and suppresses heterochromatin formation.

### TERRA depletion leads to increased ATRX abundance near transcription start sites

**Figure 2.**
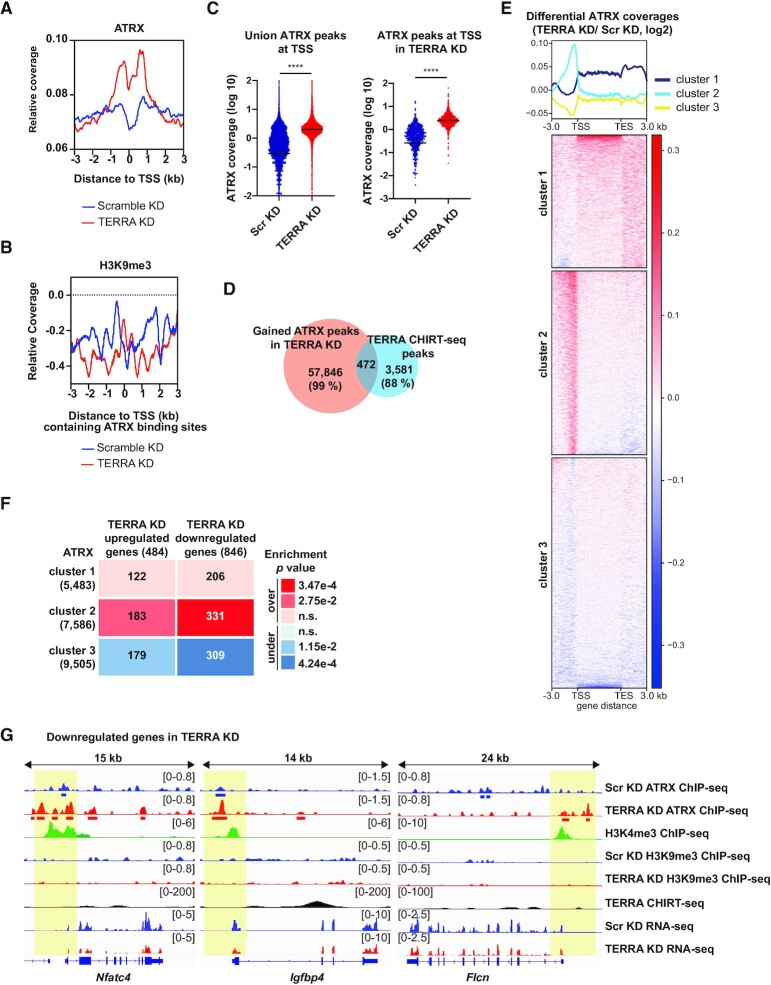
TERRA depletion increases ATRX occupancy near the transcription start sites (TSS). (**A**) Metagene profiles of ATRX coverage at TSS after TERRA depletion for 12 h in mouse ES cells. (**B**) Metagene profiles of H3K9me3 coverage at TSS containing ATRX binding sites after TERRA depletion. (**C**) Dot plots showing increased ATRX coverage at TSS (within 1kb) after TERRA depletion. All ATRX peaks (union) at TSS in both scramble KD and TERRA KD were selected (left). ATRX peaks at TSS in TERRA KD were selected (right). (**D**) Venn diagram showing the number of intersected peaks of gained ATRX peaks after TERRA depletion and TERRA binding sites. (**E**) Clustered heatmaps showing differential ATRX coverage (TERRA vs Scr ATRX density, log2) on all genes in comparison of Scr KD and TERRA KD samples. Three clusters were classified using the K-means method. (**F**) Enrichment analyses for ATRX clusters and differentially expressed genes (DEGs) after TERRA knockdown. Numbers of overlapping genes shown in the table. The over or under enrichments, p values were determined by hypergeometric distribution, and labeled with different colors in the table. (**G**) Genomic tracks displaying an increased ATRX occupancy near the TSS of downregulated genes *Nfatc4, Igfbp4, and Flcn* after TERRA depletion. No enrichment of H3K9me3 around TSS. H3K4me3 ChIP-seq indicating active TSS. Red and blue bars, ATRX peaks called by MACS.

Interestingly, metagene profiles showed that ATRX was raised near TSS in TERRA KD cells in comparison to scramble KD cells (Figure 2A). We then asked whether ATRX recruitment to TSS is related to H3K9me3 formation. ATRX binding sites near active TSS after TERRA depletion were selected for metagene analysis (Figure 2B). In contrast to the subtelomeric regions, H3K9me3 coverages were under-represented (signals below the basal level) at active TSS containing ATRX binding sites in both scramble and TERRA knockdown cells (Figure 2B), indicating that the recruitment of ATRX at TSS in TERRA knockdown cells is not accompanied with H3K9me3 formation. The annotation of ATRX peaks based on genomic features depicted that gained ATRX peaks after TERRA depletion were located across various regions of the genome, with the highest ratio of increase (12-fold) in promoter-TSS regions (Supplementary Figure S2A). Dot plots also displayed a significant elevation of ATRX coverage at TSS after TERRA depletion (Figure 2C).

Next, we tested whether the gained ATRX peaks near TSS after TERRA depletion contain TERRA binding sites. By overlapping unique ATRX peaks in TERRA knockdown cells with TERRA CHIRT-seq peaks (31) (Figure 2D), 99% of the gained ATRX peaks were found outside of TERRA binding sites (Figure 2D). Only a small fraction (less than 1%) of gained ATRX peaks in the promoter-TSS regions contained TERRA binding sites (Supplementary Figure S2B), indicating that increased ATRX occupancy at TSS is not often coupled with TERRA binding sites.

### Elevation of ATRX abundance near TSS is associated with differentially expressed genes after TERRA depletion

To test if elevated ATRX abundance at TSS is corresponding to gene expression changes after TERRA depletion, we performed a clustering analysis of differential ATRX coverage by comparing TERRA KD and scramble KD ATRX ChIP-seq data for all genes and classified three clusters by the *k*-means method as shown in the heatmap (Figure 2E). Genes in cluster 2 displayed an elevation of ATRX coverage around TSS in TERRA knockdown cells, while genes in cluster 1 and cluster 3 showed either no changes or depletion (Figure 2E). Gene expression profiles were obtained from RNA-seq data after TERRA knockdown for 12 h (31). Comparing differentially expressed genes (DEGs), genes in cluster 2 with elevated ATRX coverage at TSS exhibited an over-enrichment with significantly upregulated or downregulated genes in TERRA knockdown cells (Figure 2F). Genes in cluster 1 and cluster 3 showed no enrichment with either upregulated genes or downregulated genes. Genome browser views provided examples of cluster 2 genes: ATRX abundance was elevated near TSS of *Nfatc4, Igfbp4*, and *Flcn* genes that were downregulated upon TERRA ablation (Figure 2G). Notably, the elevation of ATRX was not accompanied by H3K9me3 coverages near TSS (Figure 2G). These results denote that increased ATRX occupancy at TSS possesses a higher frequency to alter gene expression after TERRA depletion.

Gene ontology analysis revealed that gained ATRX peaks near TSS in TERRA KD cells were enriched in genes involved in the regulation of transcription, stem cell maintenance, intracellular signal transduction, neuron differentiation, brain development, apoptosis, and cell cycle, while lost ATRX peaks in TERRA KD were enriched in genes involved in neuron projection, phosphorylation, cytoskeleton anchoring at plasma membrane and protein homooligomerization (Supplementary Figure S2C).

### TERRA maintains DNA G-quadruplex structures

**Figure 3.**
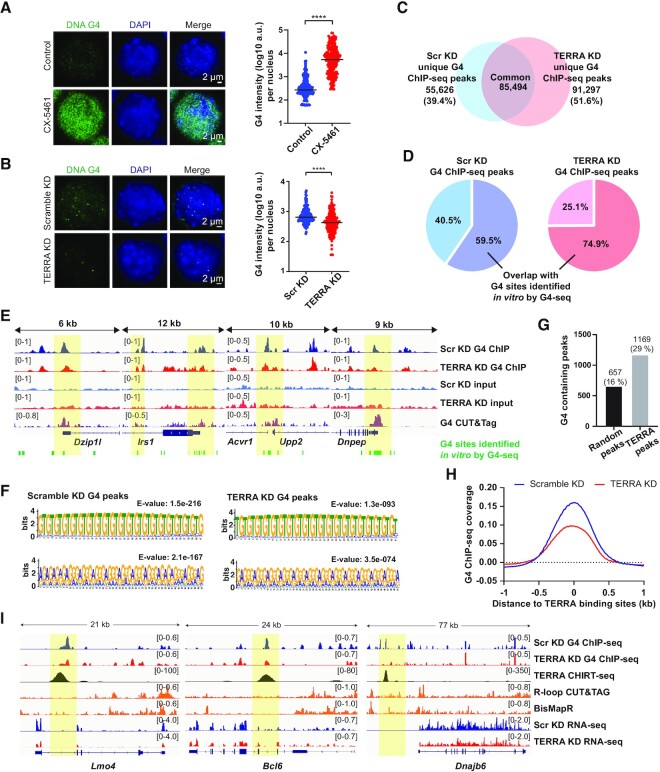
TERRA promotes DNA G-quadruplex formation. (**A**) DNA G4 immunostaining in mES cells (left panel) treated with CX-5461, a G4 stabilizer. Quantification of G4 intensity per nucleus (right panel). n > 200. bars, median. **** *P* < 0.0001, by Mann–Whitney U test. (**B**) DNA G4 immunostaining (left panel) at 6 hrs after TERRA knockdown by ASOs in mES cells. G4 intensity is reduced after TERRA depletion. Quantification of G4 intensity per nucleus (right panel). Three independent experiments showed similar results. n > 200. Bars, median. **** *P* < 0.0001, by Mann–Whitney *U* test. (**C**) Number of G4 ChIP-seq peaks shared in scramble KD and TERRA KD cells. (**D**) Percentage of G4 ChIP-seq peaks overlapping with G4 sites identified *in vitro* by G4-seq. (**E**) Genomic browser views of G4 ChIP across the indicated genes. Yellow shades highlighting the regions of G4 ChIP-seq peaks overlapping with G4 CUT&Tag and G4 sites identified *in vitro* by G4-seq. (**F**) Motif analysis for G4 ChIP-seq using MEME shows dominant motifs containing G-rich sequences. (**G**) Bar graph presenting the number of TERRA binding sites containing G4 ChIP-seq peaks. The same number of random peaks (*n* = 4052) were selected as a control and overlapped with G4 ChIP-seq peaks. (**H**) Metaplots of G4 density at TERRA binding sites. G4 coverage at TERRA binding sites is reduced after TERRA depletion. (**I**) Genomic browser views of G4 abundance at the TERRA binding sites. Yellow shades highlighting the reduced G4 signals at TERRA binding sites upon TERRA depletion. R-loop profiles from R-loop CUT&Tag and BisMapR.

Given that ATRX was found to be involved in DNA G4 formation *in vivo* (42,43), we asked whether TERRA depletion impacts G4 structures. We performed immunostaining for DNA G4 structures using the BG4 antibody. To test the sensitivity of BG4 staining, cells were treated with CX-5461, a DNA G4-stabilization ligand to induce G4 formation (58). RNase A was treated prior to DNA G4 immunostaining to eliminate RNA signals (Supplementary Figure S3A). DNA G4 immunostaining revealed a significant increase in DNA G4 signals in cells treated with CX-5461 (Figure 3A). Remarkably, we observed a significant decrease of DNA G4 intensity in TERRA knockdown cells compared to control cells (Figure 3B), suggesting that TERRA sustains DNA G4 formation.

To study DNA G4 structures *in vivo* on a genome-wide scale, G4 ChIP-seq was conducted using BG4 antibody. Two biological replicates showed a high correlation in scatter plots (Supplementary Figure S3B), with Pearson's *r* values 0.70 and 0.87 for scramble KD and TERRA KD respectively, indicating the reproducibility of G4 ChIP-seq. More than 100 thousand G4 peaks were identified and approximately 85 thousand peaks were shared in TERRA and scramble KD cells (Figure 3C). G4 coverage was enriched over genes such as promoter regions, untranslated regions (UTR), and exons (Supplementary Figure S3C). To validate the G4 ChIP-seq data, we compared G4 ChIP-seq with G4-seq (6), which identifies G4 structures *in vitro* based on DNA polymerases pausing at G4 forming sites that are stabilized by G4 ligands during polymerase chain reaction. Approximal 60∼75% G4 ChIP-seq peaks contained G4 sites identified by G4-seq (Figure 3D). G4 ChIP-seq peaks at TSS were also highly overlapped with G4 CUT & Tag peaks (59) (Figure 3E, Supplementary Figure S3D). The top two enriched motifs of G4 ChIP-seq peaks identified by MEME Suite (Motif-based sequence analysis tools) were TG repeats and GGGA repeats (Figure 3F) in both scramble and TERRA KD cells. GGGA repeats are composed of canonical G4 forming sequences, confirming that G4 ChIP-seq identifies G4 forming sequences.

We sought to explore whether TERRA binding sites consist of G4 structures. We intersected the TERRA binding sites obtained from TERRA CHIRT-seq (31) with G4 ChIP-seq peaks and found that ∼29% of TERRA binding sites shared with G4 peaks (Figure 3G), while only 16% of random sampling peaks overlapped with G4 peaks. Remarkably, the metaplot displayed a reduction of G4 coverage at TERRA binding sites (Figure 3H) upon TERRA depletion, implying that TERRA promotes G4 formation at TERRA target sites. To test if TERRA forms R-loop structures at TERRA binding sites that are accompanied by G4 structures, we compared R-loop profiles obtained from CUT&Tag (59) and found that about 20% of TERRA binding sites contained R-loop peaks (Supplementary Figure S3E). Among them, 85% of TERRA binding sites containing R-loops overlapped with G4 peaks. 20% of TERRA binding sites that overlapped with G4s and R-loops contained telomeric repeat sequences, and some examples were found in the intergenic regions (Supplementary Figure S3E, F). 40% of TERRA binding sites containing G4 structures had no R-loop structures (Supplementary Figure S3E). The data showed that R-loops were highly associated with G4 structures at TERRA binding sites, but only a subset of TERRA binding sites held G4 and R-loop structures.

The previous report demonstrated that TERRA binding density was highly enriched over downregulated genes after TERRA depletion (31), indicating that TERRA may bind to these genes to regulate gene expression. Genome browser views presented several downregulated genes such as *Lmo4*, *Bcl6* and *Dnajb6* that contained the reduction of G4 coverage at TERRA target sites within the gene body or upstream (Figure 3I), where R-loops were not enriched.

### TERRA promotes G-quadruplex formation around transcription start sites

**Figure 4.**
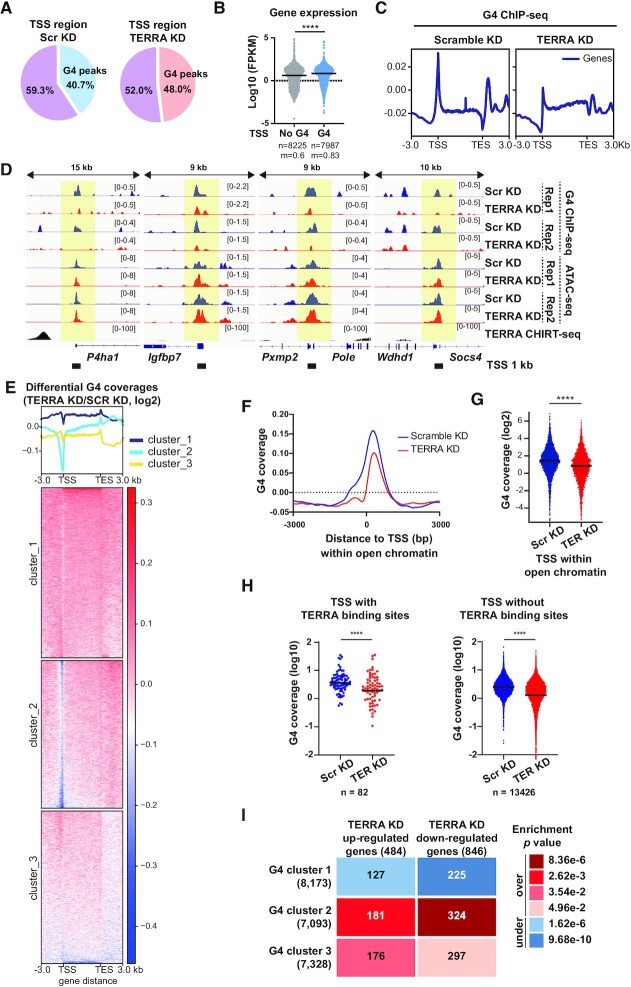
Reduced G4 formation near transcription start sites is associated with DEGs after TERRA depletion. (**A**) Percentage of transcription start sites (TSS) containing G4 ChIP-seq peaks. (**B**) The expression levels, shown in fragments per kilobase of transcripts per million mapped reads (FPKM), of genes containing G4 peaks or no G4 peaks within 1 kb of TSS. Each dot represents one gene. P value, Mann–Whitney *U* test. n, number of genes. m, median values. (**C**) Metagene analyses of G4 coverage over genes after TERRA depletion. (**D**) Genomic browser views showing that G4 abundance at TSS is reduced after TERRA depletion. G4 signals overlap with ATAC-seq peaks at TSS. (**E**) Clustered heatmaps showing differential G4 coverage (TERRA versus Scr G4 density, log_2_) on all genes in comparison of Scr KD and TERRA KD samples. Three G4 clusters were classified using the K-means method. (**F**) Metagene analyses of G4 coverages at TSS within accessible chromatin. Regions selected from TSS (±3kb) containing the overlapping of G4 ChIP and ATAC-seq peaks. (**G**) Dot plots showing G4 coverages at active TSS (±3 kb of TSS) containing G4 peaks within accessible chromatin. Bars, mean. **** *P* < 0.0001, Wilcoxon paired signed-rank test. Regions were selected from TSS (±3 kb) containing H3K4me3, G4 and ATAC-seq peaks. (**H**) Dot plots showing G4 coverage at TSS (±3 kb) containing TERRA binding sites or no TERRA binding sites. Bars, mean. **** *P* < 0.0001, Wilcoxon paired signed-rank test. (**I**) Enrichment analyses for G4 clusters and DEGs after TERRA knockdown. Numbers of overlapping genes shown in the table. The over or under enrichments and *P* values were determined by hypergeometric distribution.

Analyzing all TSS in mouse ES cells, above 40% of TSS regions contain G4 peaks (Figure 4A), highlighting the role of G4 structures in regulating gene expression. To examine whether G4 peaks at TSS are correlated with transcription activity, we compared G4 ChIP-seq and RNA-seq data. Genes containing G4 peaks within 1kb of TSS displayed higher gene expression levels in comparison to genes without G4 peaks (Figure 4B) in mouse ES cells. The data supports the idea that G4 peaks are associated with active genes (7,8).

Interestingly, metagene profiles showed that G4 densities decreased around TSS after TERRA depletion (Figure 4C, E). In addition, G4 peaks partially overlapped with ATAC-seq peaks, and the overlapped peaks occupied 43% of TSS (Figure 4D, Supplementary Figure S4A). TERRA depletion exhibited a decline of G4 abundance at chromatin accessible sites determined by ATAC-seq (Supplementary Figure S4B, left panel), whereas the chromatin accessibility at these G4 sites was not altered (Supplementary S4B, right panel). Analyses of G4 coverages at TSS within open chromatin indicated that TERRA depletion leads to a significant reduction of G4 signals at TSS (Figure 4F, G), suggesting that TERRA maintains G4 structures, particularly near transcription start sites.

Next, we asked whether the reduction of G4 at TSS is accompanied by TERRA binding sites. Dot plots showed that reduction of G4 coverage occurs at TSS containing TERRA binding sites and also at TSS without TERRA binding sites (Figure 4H) after TERRA depletion. Similarly, TERRA depletion results in G4 reduction in non-TSS regions (Supplementary Figure 4C). The number of TSS without TERRA binding sites is extremely higher than the number of TSS with TERRA binding sites (Figure 4H), suggesting that TERRA-mediated DNA G4 maintenance is not mainly dependent on TERRA binding at the same sites.

### G4 reduction around TSS is associated with differentially expressed genes upon TERRA depletion

To investigate whether G4 reduction is correlated with altered gene expression after TERRA depletion, G4 coverages of all genes in scramble and TERRA knockdown cells were compared for calculating differential G4 coverages. Three clusters were classified by K-means algorithm as shown in the heatmap (Figure 4E). Genes in cluster 2 displayed a robust depletion of G4 occupancy at TSS in TERRA knockdown cells, while genes in clusters 1 and 3 showed smaller changes at TSS. In comparison with differentially expressed genes after TERRA depletion, genes in cluster 2 with depleted G4 coverage at TSS exhibited the most significant over-enrichment with dysregulated genes in TERRA knockdown cells (Figure 4I), indicating that G4 reduction near TSS is coupled with the alteration of transcription induced by TERRA depletion.

### Diminished G4 structures within the first exon–intron correlate with gene repression

**Figure 5.**
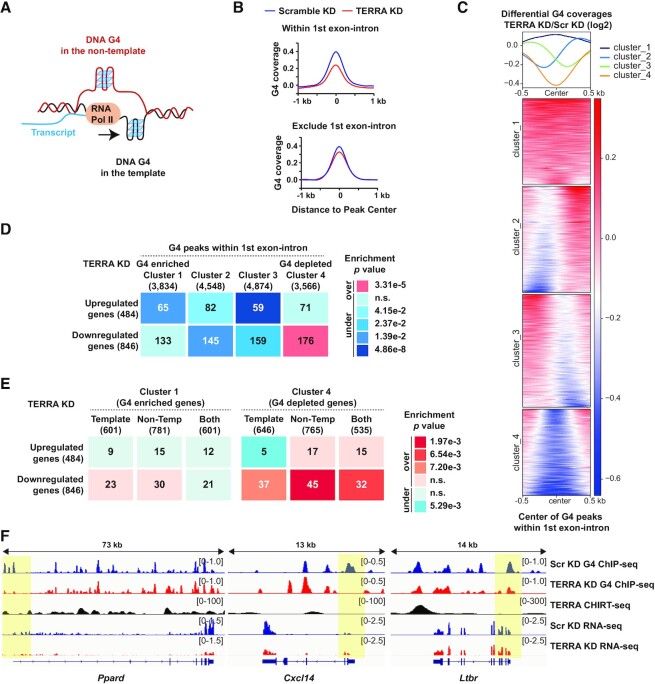
G4 abolitions within first exon/intron enrich in downregulated genes after TERRA depletion. (**A**) Illustrations of RNA polymerases encountering DNA G4 structures in the template or the non-template. (**B**) Meta-analyses of G4 ChIP-seq coverages at G4 peaks within first exon–intron (top) or at G4 peaks over gene body excluding first exon–intron (bottom). (**C**) Clustered heatmap showing differential G4 coverage (TERRA vs Scr G4 density, log_2_) at G4 peaks within first exon/intron in comparison of TERRA KD and Scr KD samples. Four G4 clusters were classified by the K-means method. (**D**) Genes containing G4 depleted signals (Cluster 4) within first exon–intron are over-enriched with downregulated genes upon TERRA depletion. Enrichment analysis for gene clusters of differential G4 coverages within first exon–intron and DEGs after TERRA knockdown. Numbers of overlapping genes shown in the table. The over or under enrichments and p values were determined by hypergeometric distribution. (**E**) Genes with G4 signals in the template, the non-template, or both were grouped. Enrichment analysis for cluster 1 and cluster 4 of differential G4 coverage within first exon–intron and DEGs after TERRA knockdown. Numbers of overlapping genes shown in the table. The enrichments and p values were determined by hypergeometric distribution. (**F**) Genome browser view of G4 reduction within first exon–intron in *Ppard*, *Cxcl14*, and *Ltbr* genes in TERRA knockdown cells. Yellow shades highlight the regions with G4 reduction within first exon–intron.

It has been postulated that unusual structures such as G-rich sequences impede RNA polymerase II movement, thereby interfering with transcription activity (60) (Figure 5A). Observations from *in vitro* transcription assays revealed that potential G4 forming sequences in the template blocks transcription; by contrast, G4 forming sequences in the non-template increase mRNA production and ensure a successful R-loop during transcription (17). To elucidate if G4 structures in the template or in the non-template are involved in gene expression changes, G4 ChIP-seq peaks within the first exon and intron were selected for metagene analysis. Metagene profiles showed that TERRA depletion led to a decline of G4 density within the first exon–intron region (Figure 5B, top), whereas the effects on other regions of the gene body were moderate (Figure 5B, bottom). Similar to the G4 analyses at TSS, genes with reduced G4 density in the first exon–intron region (cluster 4) after TERRA depletion were clustered (Figure 5C), and showed an over-enrichment with downregulated genes (Figure 5D). To identify G4 structures appearing in the template or non-template, G4 strand information was obtained from *in vitro* G4-seq (6) and integrated into G4 ChIP-seq data. Three groups were separated: genes containing G4-depleted signals in the template, the non-template, or both the template and the non-template. All three groups displayed an over-enrichment with downregulated genes after TERRA depletion (Figure 5E), with the most significant enrichment in the non-template. The results demonstrate that the reduction of G4 structures within the first exon–intron is more associated with gene repression than other gene clusters upon TERRA depletion (Figure 5D). Genome browser views displayed examples of severe G4 reduction within the first exon–intron region but mild effects on other regions of the gene body (Figure 5F).

### Decreased G4 formation is coupled with increased ATRX occupancy around TSS upon TERRA depletion

**Figure 6.**
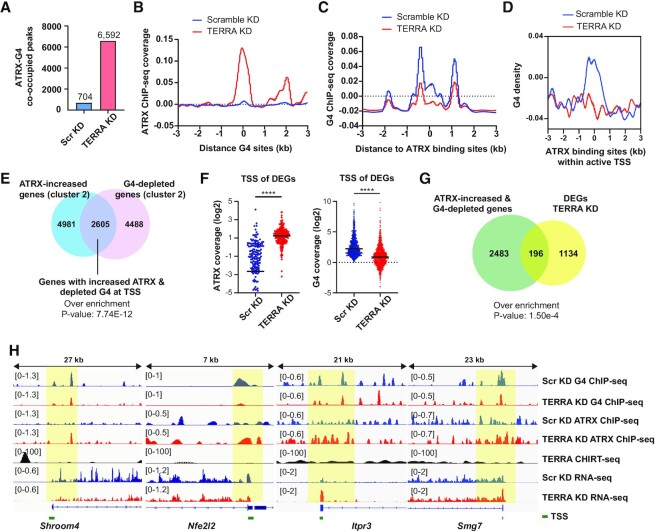
Increased ATRX occupancy is associated with DNA G4 reduction around TSS. (**A**) Numbers of ATRX ChIP-seq peaks overlapping with G4 ChIP-seq peaks. TERRA depletion increases co-occupied peaks of ATRX and G4. G4 ChIP-seq peaks selected from scramble KD. ATRX ChIP-seq peaks selected from either scramble KD or TERRA KD. (**B**) Metagene analyses of ATRX ChIP-seq showing that ATRX occupancy increases at G4 sites after TERRA depletion. G4 sites (G4 ChIP-seq peaks) selected from scramble KD. (**C**) Metagene analyses of G4 ChIP-seq showing reduced G4 signals at ATRX binding sites after TERRA depletion. ATRX binding sites (ATRX ChIP-seq peaks) selected from TERRA knockdown. (**D**) Metagene analyses of G4 ChIP-seq showing reduced G4 density at ATRX binding sites within active TSS. Regions selected by shared peaks of H3K4me3 ChIP-seq at TSS and ATRX ChIP-seq in mES cells. ATRX binding sites selected from TERRA knockdown. (**E**) Number of overlapping genes between ATRX cluster 2 (increased ATRX at TSS in TERRA KD) and G4 cluster 2 (reduced G4 at TSS in TERRA KD). P value, hypergeometric test. ATRX cluster 2 showing an over-enrichment with G4 cluster 2. (**F**) Dot plots showing ATRX and G4 coverages at TSS (±3kb) of DEGs after TERRA depletion. **** *P* < 0.0001, Wilcoxon paired signed-rank test. (**G**) Number of overlapping genes between increased ATRX/reduced G4 signals at TSS and DEGs after TERRA depletion. *P* value, hypergeometric test. (**H**) Genomic views displaying decreased G4 and increased ATRX abundances near the TSS of downregulated genes (*Shroom4*, *Nfe2l2)* and upregulated genes (*Itpr3*, *Smg7*) after TERRA depletion.

As the loss of TERRA resulted in the elevation of ATRX and the decline of G4 occupancy at TSS, we surmise that the increased ATRX occupancy may contribute to the reduction of G4 formation. Analyzing ATRX and G4 co-occupied sites, the number of ATRX peaks overlapped with G4 peaks increased more than 9 folds after TERRA depletion (Figure 6A). Additionally, metagene profiles showed that ATRX coverage at G4 sites increased in TERRA knockdown cells compared to scramble knockdown cells (Figure 6B). Conversely, the G4 coverage at ATRX binding sites decreased after TERRA depletion (Figure 6C). When selecting ATRX binding sites near TSS, G4 coverage was robustly reduced at TSS within 1 kb in length upon TERRA depletion (Figure 6D). The results indicate that TERRA prevents ATRX from loading to G4 sites and promotes G4 formation around TSS.

Next, we asked whether genes containing depleted G4 signals at TSS commonly share with genes displaying elevated ATRX coverage at TSS after TERRA depletion. Interestingly, ATRX cluster 2 genes showed a significant over-enrichment with G4 cluster 2 genes (Figure 6E, Supplementary Figure S5A, B), indicating that genes displaying increased ATRX coverages at TSS are commonly shared with genes containing depleted G4 signals. To test if altered gene expression upon TERRA depletion is associated with the changes of ATRX and G4 occupancy, we analyzed the ATRX and G4 coverages at the TSS of differentially expressed genes. We found that ATRX coverage was increased at the TSS of DEGs, while G4 coverage was decreased (Figure 6F). Moreover, genes with depleted G4 and elevated ATRX coverages at the TSS exhibited an over-enrichment with DEGs in TERRA knockdown cells (Figure 6G, Supplementary Figure S5C). Examples of several downregulated genes (*Shroom4 and Nfe2l2*) and upregulated genes (*Itpr3* and *Smg7*) in TERRA knockdown cells displayed the pattern of increased ATRX but decreased G4 coverages near TSS (Figure 6H). Notably, TERRA binding sites are not at the TSS of these genes. These results indicate that the events of elevated ATRX and reduction of G4 at TSS occur at non-TERRA target sites.

### ATRX inhibits DNA G4 formation and contributes to gene repression upon TERRA depletion

**Figure 7.**
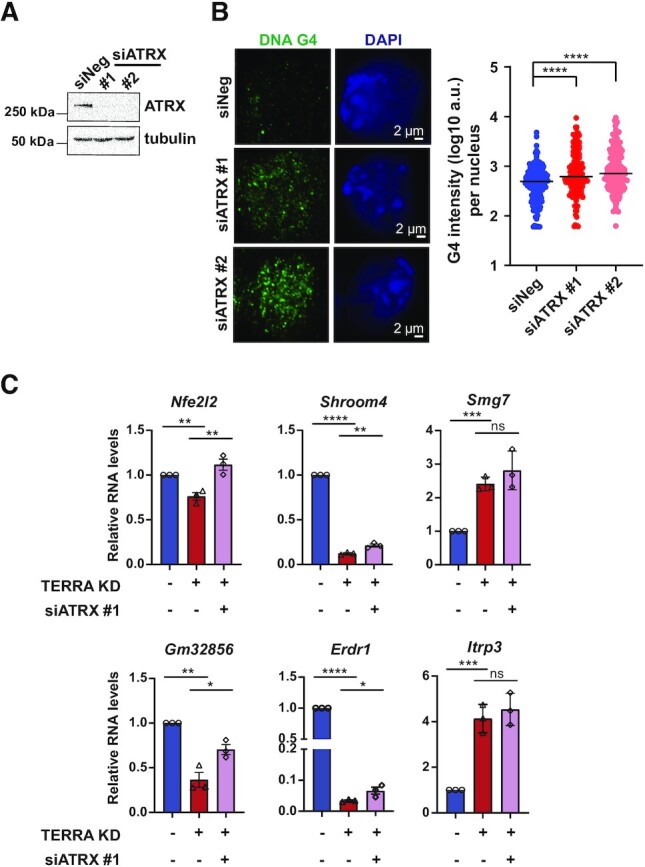
ATRX suppresses G4 formation and mediates gene repression caused by TERRA depletion. (**A**) Western blot showing ATRX protein levels after 48h knockdown using two different siRNAs in mES cells. (**B**) DNA G4 immunostaining in ATRX-depleted cells (left). Quantification of the G4 intensity in the nucleus (right). Each dot represents an individual nucleus, and a total of at least 180 nuclei were analyzed for each transfection group. Three independent experiments showed similar results. Bars, median values. **** *P* < 0.0001, by Mann–Whitney *U* test. (**C**) RT-qPCR showing the relative RNA levels after silencing ATRX in TERRA knockdown (KD) mES cells. Data from three independent experiments. Bars, mean ± SEM. **** *P* < 0.0001, ** *P* < 0.01, * *P* < 0.05, ns (no significance), by Student's *t*-test.

To further validate that ATRX regulates G4 maintenance, ATRX was silenced using two siRNAs in mES cells. DNA G4 immunostaining displayed a significant increase in G4 intensity in ATRX knockdown cells compared to control knockdown cells (Figure 7A, B), suggesting that ATRX suppresses DNA G4 formation.

To test if ATRX mediates gene dysregulation caused by the loss of TERRA, we performed double knockdown experiments in mES cells and assessed the expression of DEGs with elevated ATRX occupancy after TERRA depletion. Silencing ATRX compromised the gene repression on *Nfe2l2 and Gm32856*, while small effects were observed on *Shroom4* and *Erdr1* in TERRA deficient cells (Figure 7C). Silencing ATRX did not significantly alter gene activation on *Itpr3* and *Smg7* (Figure 7C). These results indicate that ATRX is in part involved in gene repression, and other factors also likely mediates the gene dysregulation induced by TERRA depletion.

## DISCUSSION

Here, we provide the evidence that TERRA is crucial for the proper localization of ATRX to genomic DNA and regulates G4 structures nearby TSS. TERRA directly binds ATRX and prevents ATRX from binding on repetitive sequences, including rDNA, retrotransposons, and telomeric repeats (Figure 1B–E). TERRA also regulates ATRX occupancy at subtelomeric regions (Figure 1F–H) and the DNA G4 forming sites at TSS that are away from telomeres (Figures 2A, 4E–H). Importantly, TERRA depletion causes the reduction of G4 near the TSS of a subset of genes that are accompanied by alteration of gene expression (Figure 4I). Moreover, ATRX depletion increases G4 formation in mouse ES cells (Figure 7A, B), supporting the function of ATRX in inhibiting DNA G4. These data suggest that TERRA competes with DNA G4 for ATRX binding and maintains DNA G4 structures on chromatin.

**Figure 8.**
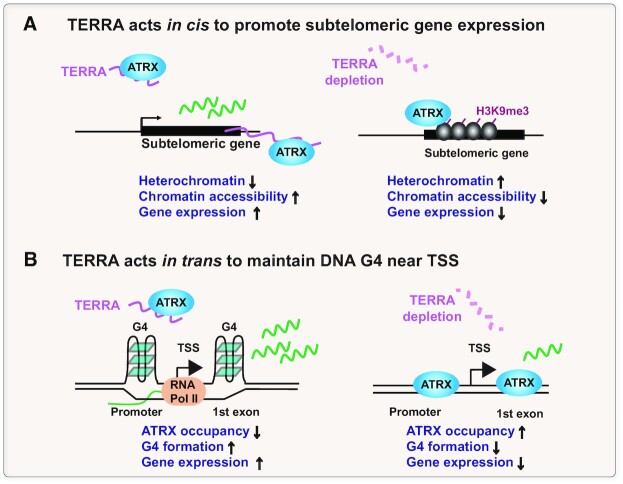
Model of TERRA modulating ATRX occupancy and G4 formation. (**A**) TERRA prevents ATRX from binding to subtelomeric regions. Increased ATRX at subtelomeric genes facilitates H3K9me3-mediated heterochromatin condensation and downregulates the expression of subtelomeric genes after TERRA depletion. (**B**) TERRA sequesters ATRX to promote G4 formation near TSS away from telomeres. TERRA depletion increases ATRX at TSS and leads to G4 DNA unwinding and gene repression.

We proposed a model (Figure 8) showing how TERRA regulates gene expression *in cis* and *in trans*. We observed a significant enrichment of ATRX and H3K9me3 on subtelomeric genes, and such enrichment leads to the compaction of subtelomeric regions and gene silencing after TERRA depletion. The results are consistent with the previous study showing that TERRA depletion causes significant downregulation of several subtelomeric genes (31). Accordingly, TERRA restrains ATRX from binding to subtelomeric regions, subsequently displacing H3K9me3 enrichment from subtelomeric regions. Our data suggest that TERRA inhibits heterochromatin formation by expelling ATRX from loading to chromatin and preventing H3K9me3 formation at subtelomeric regions, thereby regulating gene expression *in cis* (Figure 8A). Our results in mouse embryonic stem cells seem to contradict the observations showing that TERRA promotes heterochromatin formation in human cancer cell lines. Knockout of human 20q-TERRA decreases the levels of H3K9me3 and H3K27me3 (61) at telomeric repeats in U2OS cells, which lack functional ATRX. TERRA knockdown using siRNAs in HCT116 cells carrying short telomeres also shows the reduction of H3K9me3 (62) at telomeric DNA. The depletion of TRF2 increases TERRA transcripts and H3K9me3 at telomeres in HeLa cells (63). It is possible that the regulation of TERRA on heterochromatin formation at subtelomeric regions may differ in various cell lines due to the different compositions of telomeric binding proteins at telomeres.

Strikingly, the occupancy of ATRX is significantly increased at TSS upon TERRA depletion, while G4 enrichment is diminished. Since H3K9me3 occupancy is underrepresented around TSS, it indicates that H3K9me3 is not related to ATRX enrichment at TSS. The association of elevated ATRX occupancy and reduced G4 signals in TERRA deficient mES cells reasons that the recruitment of ATRX at TSS may promote the destruction of G4 at TSS. The mechanisms by which ATRX inhibits G4 formation remain elusive. The *in vitro* biochemistry assay demonstrated that ATRX failed to unwind G4 structures (64). It has been reported that ATRX is able to suppress R-loop formation *in vivo* (65) and *in vitro* (41). The ability to inhibit R-loops by ATRX may destabilize the extrusion of the G-rich single-stranded DNA, therefore suppressing G4 formation. In the absence of TERRA (Figure 8B), ATRX is no longer bound by TERRA, leading to the recruitment of ATRX to TSS. While the reduction of G4 signals at TSS could be found at TERRA target sites (Figure 4H), the majority of G4 reduction was found at non-TERRA target sites, suggesting that TERRA-mediated DNA G4 maintenance does not rely on TERRA binding at the same sites. As TERRA could be diffusible and interact with transcription regulators (31), it seems that TERRA acts *in trans* and sequesters ATRX to regulate G4 formation across the genome (Figure 8B).

The regulation of G4 structures by TERRA could involve other mechanisms. TERRA is capable of invading into double-stranded DNA, forming an R-loop at its target sites and allowing another strand of G-rich DNA to fold into a G4 structure, hence facilitating DNA G4 formation. In agreement with that, the genomic R-loop analysis shows that a subset of TERRA binding sites containing R-loops highly overlapped with G4 structures (Supplementary Figure S3E-S3F). There are also G4 depleted regions where no increased ATRX occupancy is observed upon TERRA depletion (Figure 6E), suggesting that TERRA depletion may interfere with G4 formation in an ATRX-independent manner (Figure 6E). TERRA may likely bind to other G4 regulators such as BLM and RTEL1, which are TERRA interacting proteins identified by TERRA iDRiP (31), and modulate their loading to the genome. The negative correlation between G4 structures and BLM or RTEL1 has been reported previously (64,66,67). The deficiency of BLM or RTEL1 promotes G4 formation *in vivo*, denoting that BLM and RTEL1 inhibit G4 accumulation.

As TERRA is a G-rich nuclear RNA that is found to interact with G4 binding proteins, we hypothesize that TERRA may act as a decoy for G4 regulators and prevent them from loading to genomic DNA, thereby promoting DNA G4 formation. More evidence is required to support this hypothesis. Altogether, our findings provide insights into the mechanism by which TERRA regulates gene expression *in cis* and *in trans*, and show how a G4 RNA might impact genomic G4 formation.

## DATA AVAILABILITY

ATRX ChIP-seq, H3K9me3 ChIP-seq, ATAC-seq, G4 ChIP-seq data after TERRA depletion have been deposited to the NCBI Gene Expression Omnibus (GEO), with accession number GSE194038. The following published sequencing data sets were analyzed in this study: H3K4me3 (68) (GSM4643762), *in vitro* G4-seq (6) (GSM3003547), G4 CUT&Tag (59) (GSM5259790), R-loop CUT&Tag (59) (GSM5259796), BisMapR (69) (GSM4875571), scramble KD (GSM1711930, GSM171933) and TERRA KD RNA-seq (31) (GSM1711931, GSM1711934) and TERRA CHIRT-seq in mESC (31) (GSM1711915).

## Supplementary Material

gkac1114_Supplemental_File
