# Supplementary material for: TERRA regulates DNA G-quadruplex formation and ATRX recruitment to chromatin
Source: Nucleic Acids Res. 2022 Nov 28;50(21):12217–34. doi: 10.1093/nar/gkac1114 (PMC9757062; doi:10.1093/nar/gkac1114)
Supplement: gkac1114_Supplemental_File [file gkac1114_supplemental_file.pdf]

SUPPLEMENTARY DATA

TERRA regulates DNA G-quadruplex formation and ATRX recruitment to chromatin

Ru-Xuan Tsai[1,#], Kuo-Chen Fang[1,#], Po-Cheng Yang[1,#], Yu-Hung Hsieh[1], I-Tien Chiang[1], Yunfei Chen[1], Hun-Goo Lee[2], Jeannie T Lee[2], Hsueh-Ping Catherine Chu[1,*]

[1] Institute of Molecular and Cellular Biology, National Taiwan University, No. 1 Sec. 4 Roosevelt Road, Taipei, Taiwan.

[2] Department of Molecular Biology, Massachusetts General Hospital, Department of Genetics, Harvard Medical School, Boston, MA 02114, USA

[#]These authors contributed equally

* To whom correspondence should be addressed. Tel: 886-233662487; Fax: 886-233662478; Email: cchu2017@ntu.edu.tw

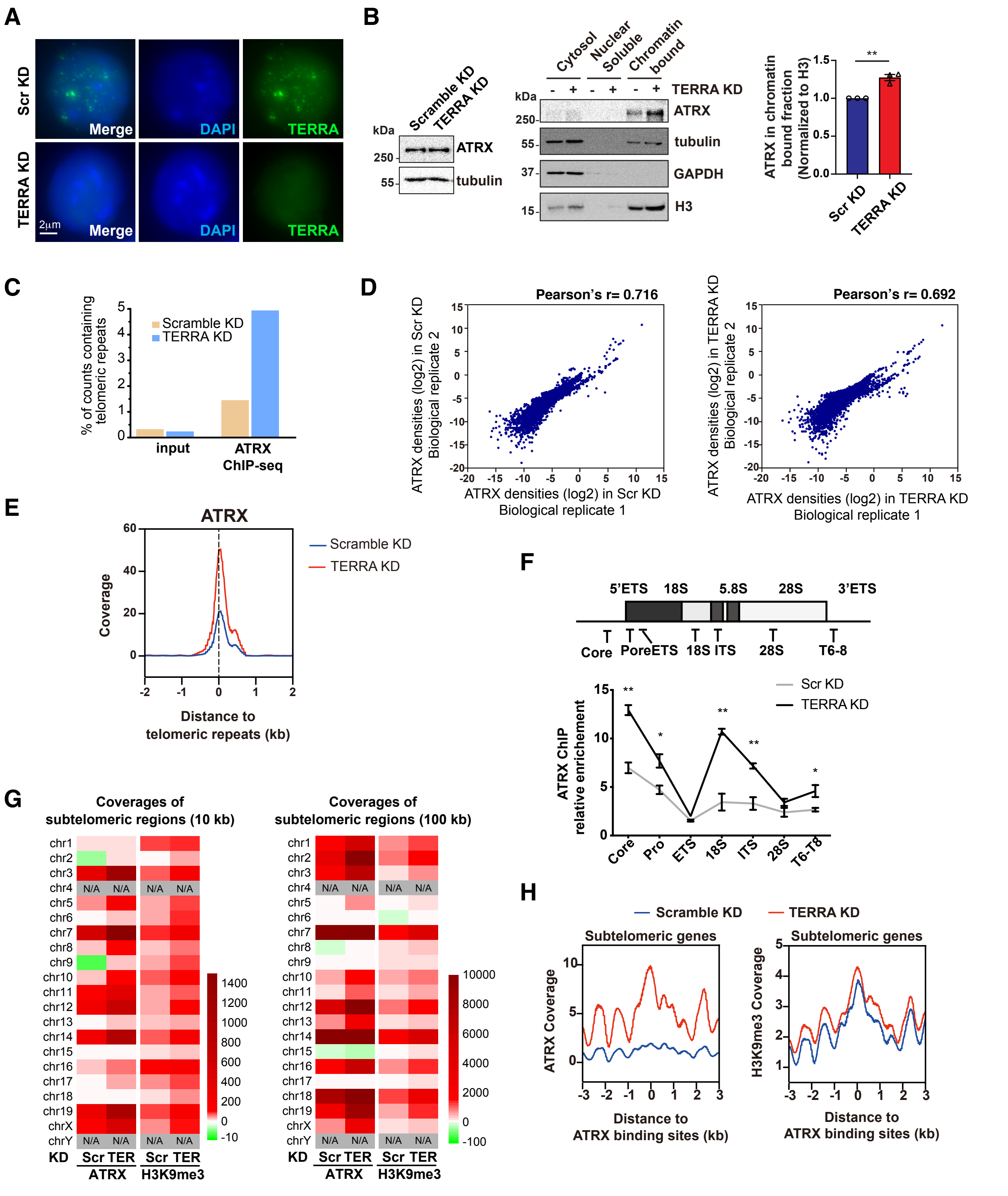

Supplementary Figure S1. TERRA depletion increases ATRX occupancy at telomeric repeats and rDNA locus.
(**A**) Mouse ES cells were transfected with antisense LNA oligos against TERRA or scramble sequences. TERRA levels were analyzed at 12-hour of post-transfection by RNA-FISH. (**B**) Western blotting to detect ATRX protein after TERRA depletion. Total cell lysates (left). Nuclear and cytosolic fractions (middle). Quantification of ATRX in chromatin bound fraction from three independent experiments (right). (**C**) Percentage of read counts containing telomeric repeats from ATRX ChIP-seq data. (**D**) Scatter plots showing the correlation between two biological replicates of ATRX-ChIP-seq. Pearson's r values shown on the top of the plots. (**E**) Metagene profiles of ATRX coverages on telomeric repeats. (**F**) Schematic diagram showing the positions of various PCR fragments across the region of the rDNA locus (top). ATRX-ChIP qPCR for rDNA locus (bottom). P values (*<0.05, **<0.01) were determined by Student's t test. Error bars, SD. (**G**) Heatmaps showing ATRX and H3K9me3 coverages at subtelomeric regions. The coverage was summed within 10 kb (left) or 100 kb (right) away from telomeric ends, and the coverages of the pure telomeric repeat tracks were excluded. (**H**) Metagene profiles of ATRX and H3K9me3 on ATRX binding sites within subtelomeric genes. ATRX binding sites were selected from TERRA knockdown cells.

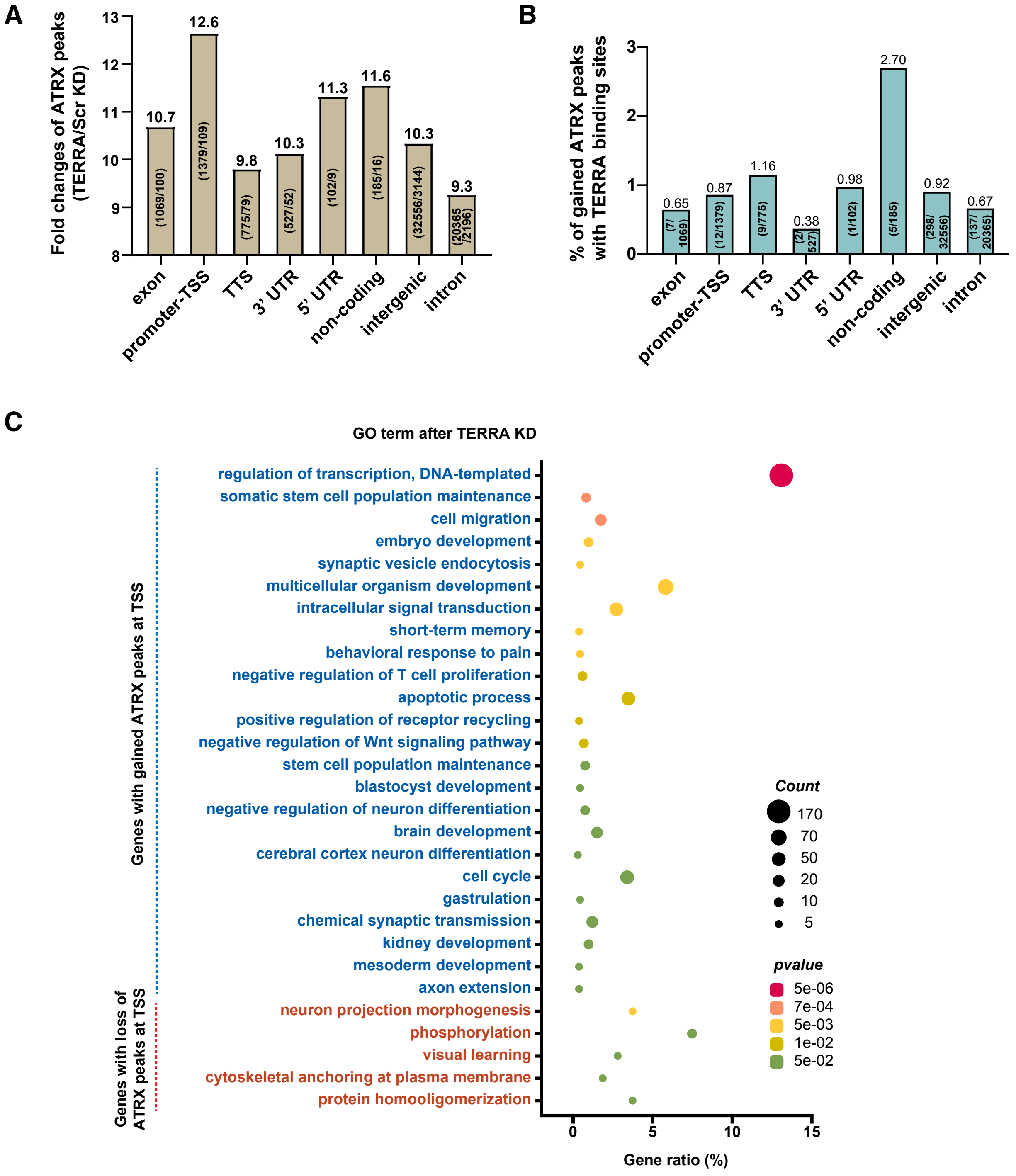

Supplementary Figure S2. Gene ontology analysis of ATRX ChIP-seq after TERRA depletion.
(**A**) Fold changes of ATRX peaks in various genomic regions after TERRA depletion. (**B**) Percentage of gained ATRX peaks containing TERRA binding sites after TERRA depletion. Numbers of gained ATRX peaks containing TERRA binding sites (common peaks of gained ATRX peaks and TERRA CHIRT-seq peaks) over gained ATRX peaks in TERRA knockdown cells. (**C**) Gene ontology (GO) analysis of biological process by DAVID bioinformatics resources for ATRX unique peaks (gained ATRX peaks) in TERRA KD or for ATRX unique peaks (loss of ATRX peaks in TERRA KD) in scramble KD cells. Genes with gained ATRX peaks (blue texts) in TERRA KD are highly enriched with biological processes involved in transcription, stem cell maintenance, brain development, and cell proliferation. Data is selected by Benjamin value less than 0.05. Genes with lost ATRX peaks (red text) in TERRA KD are highly enriched with biological processes involved in neuron morphogenesis and phosphorylation.

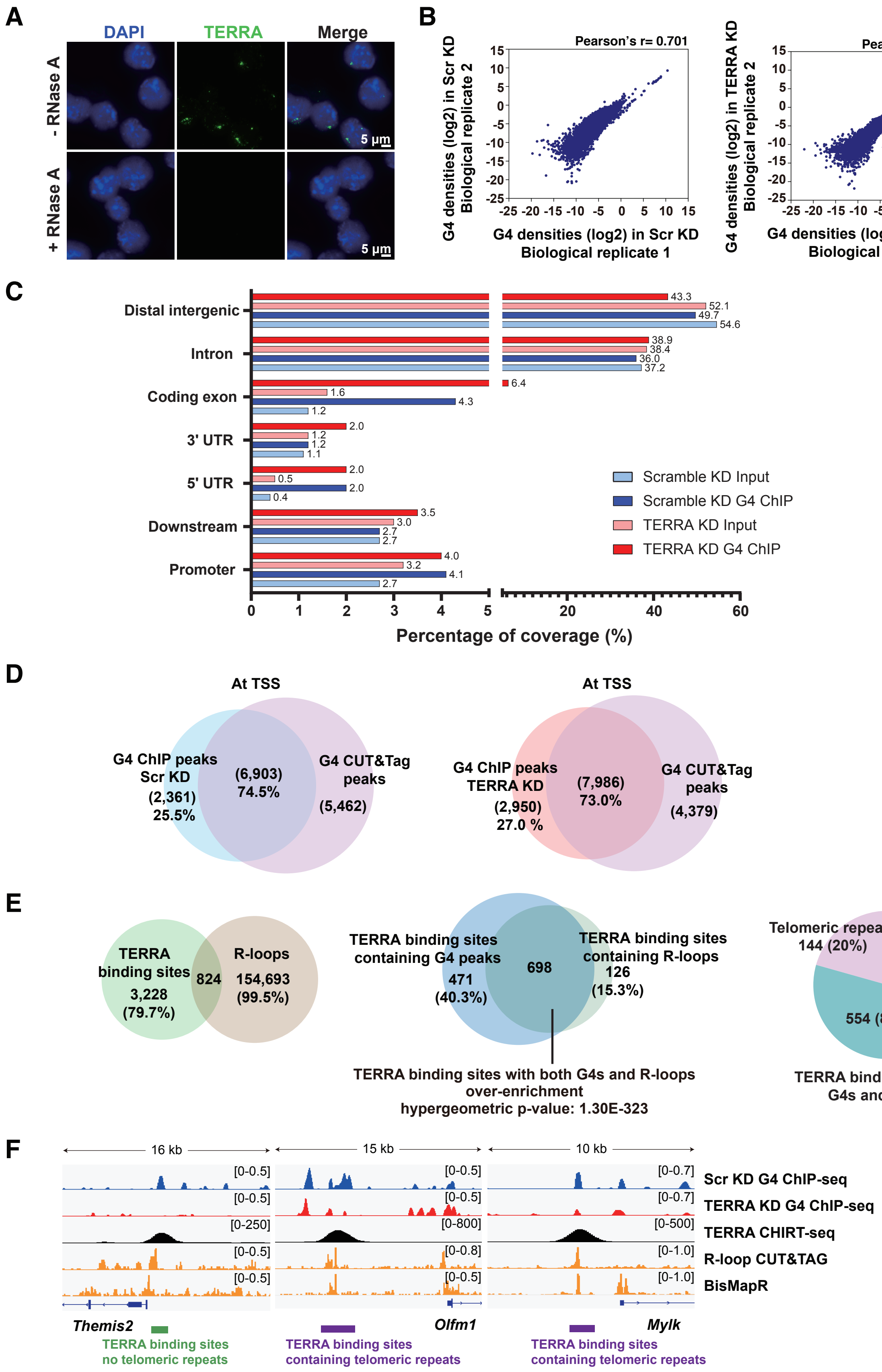

Supplementary Figure S3. G4 ChIP-seq profiling in mouse ES cells.

(**A**) RNA-FISH to detect TERRA RNA in mouse ES cells. RNase A treatment eliminated all TERRA RNA signals. (**B**) Scatter plots showing the correlation of G4 coverages between two biological replicates. (**C**) Relative enriched regions of G4 ChIP-seq analyzed by cis-regulatory element annotation system (CEAS). (**D**) Number of overlapping peaks between G4 ChIP-seq and G4 CUT&Tag peaks at TSS in mES cells. (**E**) Number of overlapping peaks between TERRA CHIRT-seq and S9.6 CUT&Tag peaks in mES cells (left). Number of TERRA binding sites containing G4s and R-loops (center). Number of TERRA binding sites containing G4s and R-loops with telomeric repeat sequence (right). (**F**) Genomic browser views of G4 abundance at the TERRA binding sites containing G4s and R-loops. R-loop profiles obtained from S9.6 CUT&Tag and BisMapR.

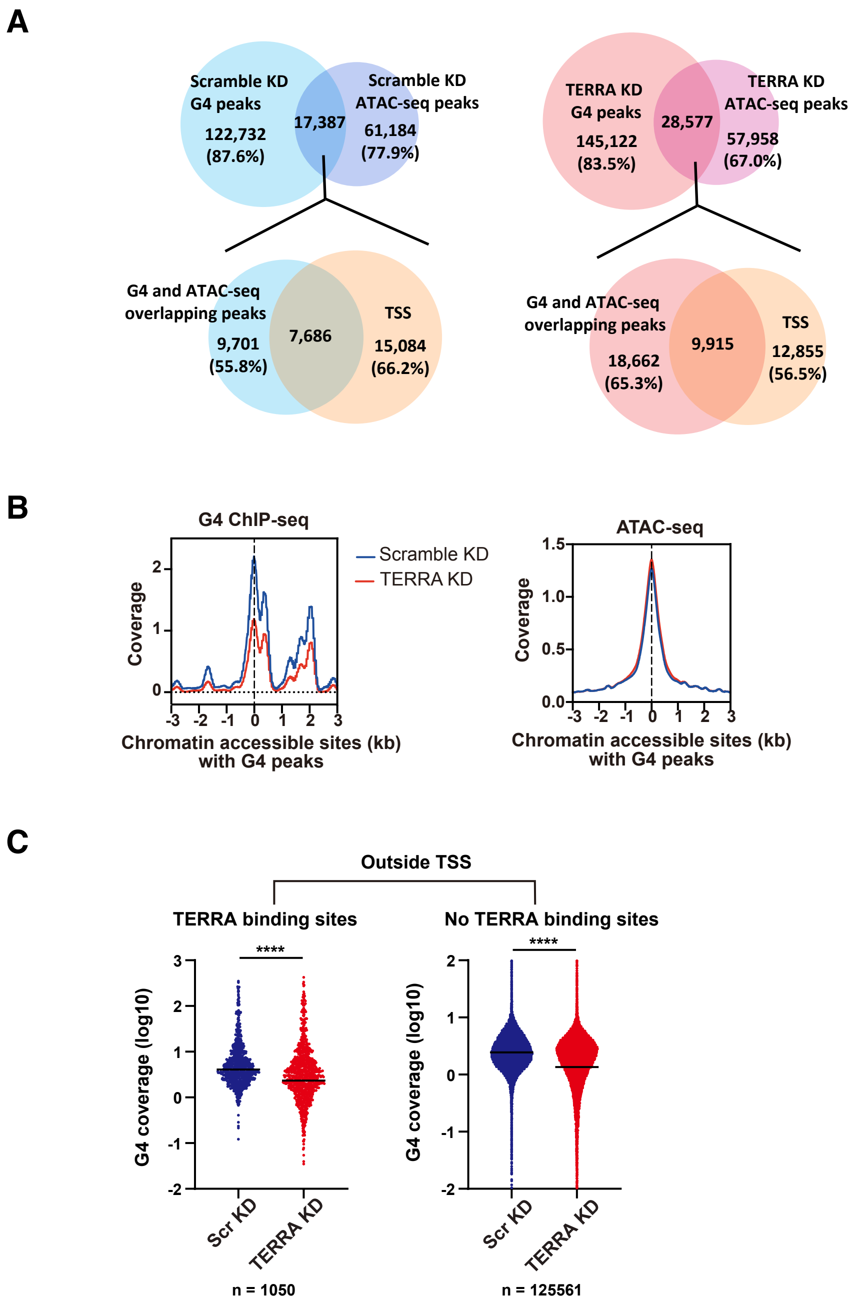

Supplementary Figure S4. Overlapping peaks between G4 ChIP-seq and ATAC-seq.
(**A**) Numbers of overlapping peaks between G4 ChIP-seq and ATAC-seq peaks in mES cells. Overlapping peaks are often located at TSS. (**B**) Metaplots of G4 ChIP-seq coverage at G4 containing open chromatin sites (left panel), which were defined by common peaks of ATAC-seq and G4 ChIP-seq. TERRA depletion decreases G4 coverage at G4 containing open chromatin sites. Metaplots of ATAC-seq coverage (right panel) at G4-containing open chromatin sites. (**C**) Dot plots showing G4 coverage outside of TSS (3kb) containing TERRA binding sites or no TERRA binding sites. Bars, mean. **** P <0.0001, Wilcoxon paired signed-rank test.

A

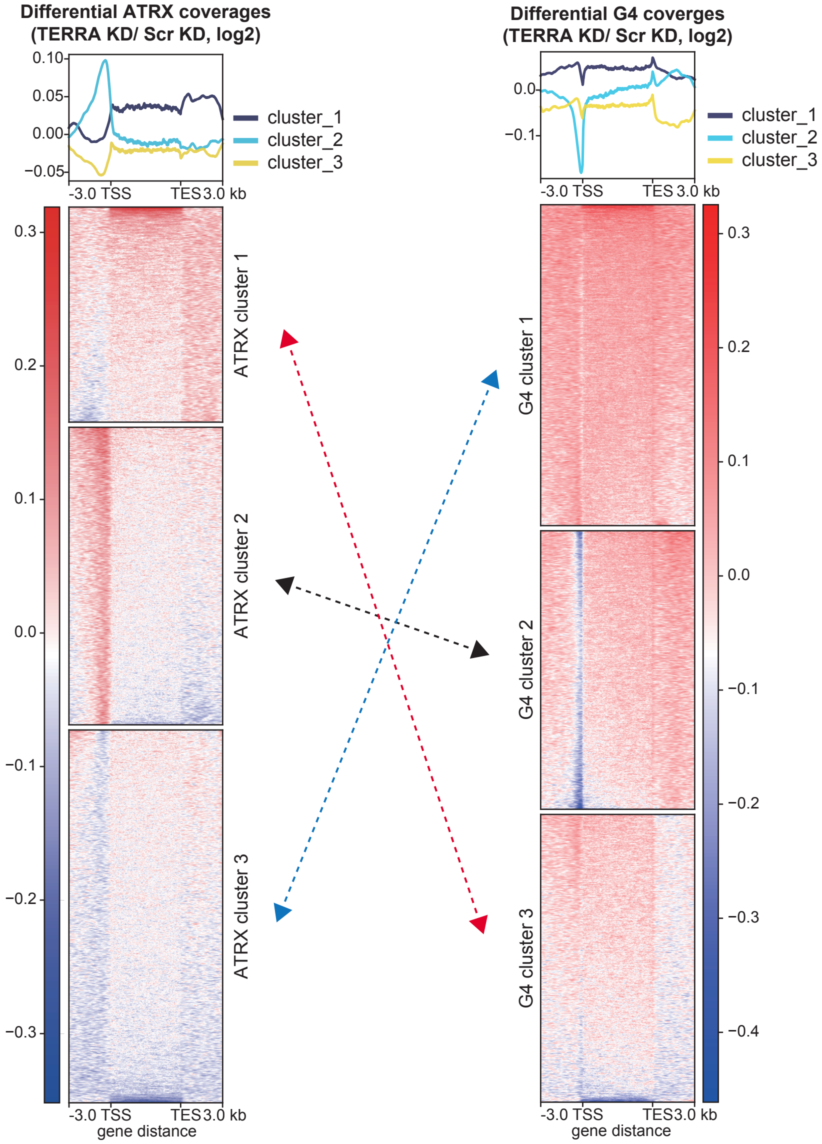

B

	ATRX cluster 1 (5,483)	ATRX cluster 2 (7,586)	ATRX cluster 3 (9,505)
G4 cluster 1 (8,173)	1,375	2,497	4,300
G4 cluster 2 (7,093)	1,778	2,605	2,691
G4 cluster 3 (7,328)	2,330	2,484	2,514

Enrichment *p* value

over: 1.04e-128; 7.79e-73; 7.70e-12; 3.02e-2; n.s.

under: n.s.; 2.00e-13; 8.25e-18; 3.54e-61; 1.53e-89

C

	TERRA KD upregulated genes (484)	TERRA KD downregulated genes (846)
ATRX cluster1 & G4 cluster3 (2330)	50	83
ATRX cluster2 & G4 cluster2 (2605)	74	122
ATRX cluster3 & G4 cluster1 (4300)	66	102

Enrichment *p* value

over: 5.23e-3; 6.91e-3; n.s.

under: n.s.; 9.20e-4; 1.80e-8

Supplementary Figure S5. Increased ATRX density at TSS is coupled with reduced G4 signals after TERRA depletion.
(**A**) Clustered heatmaps of differential ATRX coverage (TERRA vs Scr ATRX density, log2) and differential G4 coverages. Arrows with dash lines indicate a significant over-enrichment between two clusters. (**B**) Numbers of overlapping genes of differential ATRX clusters and differential G4 clusters are shown in the table. P values of over or under enrichments, hypergeometric distribution. (**C**) Numbers of overlapping genes of ATRX & G4 clusters and DEGs in TERRA knockdown. P values, hypergeometric distribution.
